# The effect of knowledge brokering on nurses’ empathy with patients receiving cardiac care: a study protocol

**DOI:** 10.1186/s12913-020-05377-1

**Published:** 2020-07-23

**Authors:** Atefeh Galehdarifard, Moloud Radfar, Mohammad Gholami, Mojgan Khademi, Farzad Ebrahimzadeh, Mohammad-Hasan Imani-Nasab

**Affiliations:** 1 Student Research Committee, Lorestan University of Medical Sciences, Khorramabad, Iran; 2 Faculty of Nursing and Midwifery, Urmia University of Medical Sciences, Urmia, Iran; 3 Social Determinants of Health Research Center, Lorestan University of Medical Sciences, Khorramabad, Iran

**Keywords:** Cardiovascular nursing, Empathy, Knowledge translation

## Abstract

**Background:**

Hospitalization could be an unpleasant experience for patients with cardiovascular disease leading to some negative emotional reactions. These emotions can be managed by nursing empathy. There are different methods for improving empathy, but some evidence indicates a dramatic drop in nurses’ empathy. In this study, we aim to provide a protocol for investigating the effect of knowledge brokering on nurses’ empathy with patients receiving cardiac care.

**Methods:**

This study protocol is developed based on SPIRIT checklist with an experimental design. The study population are nurses working in cardiac wards of three educational hospitals in western Iran, Khorramabad. The quota sampling method is used. The sample size is 100 individuals. The samples will be assigned to two intervention and comparison groups using stratified random allocation method. Permuted block randomization is used in each stratum. To prevent contamination between participants; firstly, the measurements of the comparison group is done. Knowledge brokering intervention is performed in 7 stages based on Dobbins’ knowledge translation framework 2009. Monica’s Empathy Construct Self-Rating Scale is used for measuring empathy. Statistical analyses are performed using SPSS (SPPS Inc. Chicago, Il, version 21). P value below 0.05 is considered as statistically significant.

**Discussion:**

To our knowledge, there is no similar study using an experimental design to examine the efficacy of a knowledge brokering method to improve humanistic knowledge. It helps nurses to improve their empathy in caring relationships.

## Contributions to the literature

- Common educational methods face some challenges in the implementation of empathy construct, which is a professional value and an element of ethical performance.
- Knowledge brokering can promote usage of empathy knowledge in clinical practice.
- The present study can help both the translation of empathy elements and better recognition of knowledge broker role.

## Background

In Eastern Mediterranean countries, such as Iran, mortality rate due to cardiovascular diseases is increasing [1]. Cardiovascular diseases can lead to emotional reactions such as depression, anxiety, and dependency in patients [2]. Nurses’ empathy with patients is a way to manage such negative reactions [3].

Empathy is the cornerstone of patient-centered care [4] and affects patients’ health positively [5, 6]. Empathy can lead to soothing the pain, preserving integrity [4], and having hope [7]. However, excessive empathy of medical staff can lead to negative consequences such as over-arousal [3, 5], depression [3], and compassion fatigue [5]. Thus, empathy should be expressed to yield positive outcomes and prevent negative consequences caused by excessive empathy.

While there is growing evidence that clinical empathy has been enhanced owing to various training strategies [6, 8], some studies indicate that nurses’ care is mechanical [9] and professional values such as empathy in nurse-patient relationship has been decreased [10, 11]. Possibly, the findings substantiating the effect of training on developing empathy skills are not used in practice or the training intervention by itself has not been able to fill the gap between theory and practice. Some studies indicate that the implementation of evidence-based practice is poor among nurses of different countries [12–14], including Iran [15]. Actually poor perceived knowledge of the implementation of evidence-based practice leads to poor decision-making and health policy development at different levels of the health system, including hospital wards [16].

To fill the gap between evidence and clinical practice, different knowledge translation interventions have been used [17–20]. Knowledge brokering is a dynamic and interactive intervention. Here the knowledge broker (KB) can use different knowledge translation interventions including tailored messages and educational meetings based on the needs of the participants [21]. Because Iranian nurses are faced with challenges in evidence-based practices [12], and Iranians’ behavioral, cultural, and socio-economic characteristics [15], it seems that knowledge brokering intervention can be implemented in Iranian culture. However, there is few evidence advocating the effectiveness of this approach for translating the knowledge of professional values such as empathy. The present state of evidence-based practices in the field of empathy indicates the necessity of making some interventions to translate empathy knowledge into practice and assess whether knowledge brokering brings about any changes in nurses’ empathy.

Adopting experimental design allows controlling the effect of contextual variables [22] which affect empathy. Consequently, the credibility of the findings regarding the efficacy of knowledge brokering intervention is enhanced. Practical aims of this project are sensitizing nurses to humanistic and ethical values, enhancing evidence-based practice, and investigating the effect of knowledge brokering on nurses’ empathy.

## Methods

The present study protocol is developed base on the SPIRIT checklist.

### The research question

What is the effect of knowledge brokering on nurses’ empathy with patients receiving cardiac care?

### Aim

To determine the effect of knowledge brokering on nurses’ empathy with patients receiving cardiac care.

### Hypothesis

Knowledge brokering brings about changes in nurses’ empathy with patients receiving cardiac care**.**

### Design

The study design is experimental (see Fig. 1: Additional file 1).

### Setting

The study is conducted in two cardiac units, three coronary care units, one intensive care unit of open heart surgery, and one angiography ward for three educational hospitals. Nurses who work in the mentioned wards use case method delivering care. This method provides more time for developing an empathic relationship. However, some researches performed in Iran illustrated lack of empathy in nurses [9]. Although ethics principles and communication skills are a part of educational curriculum of nursing students in Iran, the empathy subject has not been defined specifically. Lack of required knowledge regarding empathy can be the reason of paucity of empathy in nurses.

### Eligibility criteria for study centers

- Educational hospitals with cardiac wards.

### Characteristics of participants

#### Inclusion criteria

- At least a bachelor’s degree in nursing.
- Working as a nurse in one of the cardiac wards under investigation.

#### Exclusion criteria

- Changing workplace to non-cardiac wards and leaving nursing profession during the study.
- Participating in other studies and training courses on empathy and effective relationship

### Sample size

The size of the primary sample is calculated using the following formula [23]:

$$ n=\frac{{\left({z}_{1-\frac{\alpha }{2}}+{z}_{1-\beta}\right)}^2\left({s^2}_1+{s^2}_2\right)}{d^2} $$

In each group, 45 participants are calculated where;

$$ {z}_{1-\frac{\alpha }{2}}=1.96\to \left(\alpha =0.05\right) $$

$$ {z}_{1-\beta }=0.84\to \left(\beta =0.20\right) $$

$$ \mathrm{S}1=\mathrm{S}2\cong \frac{R}{6}=\kern0.5em 84 $$

$$ d=50 $$

“S1” and “S2” are the standard deviations of Monica’s empathy scores in the both groups which are considered as being equal here. The minimum difference between the mean of empathy scores which is significant for the researcher is “d”. With a 10% possibility of participants’ loss, the final sample size in each group is considered as 50.

### Sampling method

Proportional to size, quota sampling is used [24]. The strata include wards under study. In each stratum, based on eligible nurses’ entrance to the study setting, convenient sampling method is used until the sample size is completed.

### Randomized allocation

After completing participants’ enrollment by KB, a list of eligible nurses coded by English alphabet is given to the statistical consultant. Using a computer-generated random number table and stratified *random* allocation techniques, the statistical consultant assigns the eligible participants to parallel groups of A and B in a 1:1 ratio according to the superiority framework. The strata are created based on gender, work experience, ward and education. In each stratum, the permuted block randomization technique is used. Then, the convenient sampling method is used in each stratum. After sampling, one of the labels of A or B is randomly selected as intervention group by flipping a coin. According to the random allocation method, two groups are matched in terms of gender, education, work experience, and ward.

### Blinding

Due to the nature of intervention, there is no blinding. However, to avoid any bias, the statistical consultant should not be aware of the nurses’ identity. Participants will not have access to the random assignment mechanism.

### Solutions for decreasing participants’ dropout rate

Establishing a friendly and trust-based relationship with nurses, providing organizational support through negotiating with intra-organizational policy makers, providing certificate of participation, offering small gifts, and holding free workshops could be common ways of promoting cooperation. Furthermore, conducting a needs assessment for developing educational content and achieving a consensus about the method, time, and place of exchanging knowledge will increase participants’ compliance and prevent their drop out. Finally, compiling a list of demographic information of the participants who dropped out along with their drop out reason will pave the way for finding an appropriate solution for preventing more participant loss.

### Concomitant interventions

Knowledge brokering is the only intervention which is performed to investigate the changes in the empathic behaviors of cardiac nurses.

### Outcomes

Empathy is measured using Persian version of Monica’s empathy questionnaire which has a Kappa coefficient of 0.888 and a Cronbach’s alpha of 0.905 [25]. Due to the nature of intervention and preventing sample contamination, at first, the assessments of the comparison group are performed. In the comparison group, the mean empathy score is calculated three times via one pretest and two posttests. To determine changes in the comparison group, we use the information obtained from one of the posttests which is closer to the intervention group for the time span between the two measurements before and after the intervention. In the intervention group, the mean empathy score is calculated three times via two pretests and one posttest. The first pretest is simultaneous with the pretest of comparison group. The second pretest is performed immediately before starting the intervention. The rationale for this decision is equating the time interval between the pretest and posttest in the both groups. The posttest is performed immediately after intervention (see Fig. 1: Additional file 1).

### Study timeline

At first time point (T1), enrollment, random allocation, and baseline assessments (pretests) are performed. At weeks 7 and 14 (T2&T3), two posttests are performed. The second pretest in the intervention group is performed at week 15 (T4). The intervention is performed for 6 to 12 weeks. Given that the amount of evidence is determined by educational needs assessment and participants’ preferences, we do not know the amount of required evidence and consequently, the time needed for translation of empathy knowledge. Hence, 12 weeks is selected as the maximum time required for performing the intervention. The posttest of the knowledge brokering group is possibly performed at weeks 28 (T5). (Refer to Table 1: Additional file 2).

### Data collection methods

The demographic data of participants is collected using a self-constructed questionnaire. To measure empathy, the Persian translation of Monica’s Empathy Construct Self Rating Scale is used. This instrument comprises 84 items. Responses are graded on a 6-point Likert scale (with − 3: extremely unlikely, and + 3: extremely likely) [26, 27]. This instrument, with a high internal consistency, split-half reliability, and high test-retest reliability is developed for nurses [28].

### Interventions

The comparison group is investigated without receiving any intervention. In the intervention group, knowledge brokering is performed based on Dobbins’ knowledge translation framework to link research producers and research users. In this framework, Dobbins describes activities which are performed by the KB in seven stages including initial and ongoing needs assessments; scanning the horizon; knowledge management; Knowledge translation and exchange; network development, maintenance, and facilitation; facilitation of individual capacity development in evidence informed decision making; and facilitation of and support for organizational change [21].

#### Characteristics of KB

KB, a master student in nursing, speaks Persian, has more than 2 years of clinical experience, has worked on empathy and knowledge translation for more than 2 years, has the experience of interpreting research findings, and has effective training skills. Through face-to-face meetings, in-person appointments, phone calls, electronic communications, and training sessions KB transfers the knowledge regarding empathy to clinical nurses and hospital managers, including head nurses, educational and clinical supervisors, and matrons. If necessary, KB is assisted by the research team including two assistant professors of nursing (M.KH & M.GH) and one assistant professor of psychiatric nursing (M.R), who have considerable clinical, educational, and research experience, and one assistant professor of health policy making (MH.I) working on evidence-based decision making.

#### The stages of translation of empathy knowledge

The intervention is performed in 6 to 12 weeks and in 7 stages. These stages are explicated as follows.

- 1. Individual assessment: educational needs assessment on empathy

Through a quantitative researcher-made questionnaire, which is a set of multiple choice questions and modelled on Dobbins’ study and census, knowledge level, educational expectations and needs of the intervention group and managers are determined. Moreover, their skills for gaining the required content about empathy is identified. In addition, the content validity of the questionnaire is checked by three nursing professors. The qualitative needs assessment continues during both individual and group interactions through informal interviews and asking open- ended questions such as “ Is there any question?”, “ Is there anything you want to know more about?”

- 2&3. Scanning the horizon and knowledge management.

KB will constantly search and review scientific evidence required for the intervention group. Furthermore, using Google Scholar alerts, KB is informed of the most recent publications on empathy and can share the obtained evidence with participants. End Note software is used to manage obtained evidences.

- 4. Knowledge translation and exchange

To achieve knowledge translation and exchange, the following actions are performed in parallel.

- 4.1. Encouraging the intervention group to search and study the evidence

Immediately after performing the second pretest and needs assessment, KB provides the intervention group with access to some academic databases (e.g., PubMed, Web of Science, Google Scholar, Scopus, etc.). Accordingly, the intervention group can independently extend their individual knowledge regarding empathy. Intervention group members are asked to record the number of their referrals to databases, the characteristics of databases and the characteristic of the article they study and report them weekly to KB.

- 4.2. Sending tailored and targeted messages

KB extracts the required knowledge on empathy from articles, databases, and if required, from books and synthesizes tailored and targeted messages. The accuracy, precision, and comprehensiveness of these messages are examined by other members of the research team. KB will weekly send tailored messages in a Telegram group entitled “empathy knowledge translation” and, if required, in private pages for 4 to 8 weeks. Telegram is a free messaging application, such as WhatsApp and Viber, mostly used by Iranians.

- 4.3. Holding training sessions

The developed training content is presented by KB in 4 to 8 training sessions held weekly for 4 to 8 weeks with sending tailored messages. In these sessions, the scientific findings are presented and discussed. Moreover, individuals’ attitudes about empathy are discussed. Each session takes about 1.5 to 2 h.

- 4.4. Individual and three-person groups interaction

In the knowledge exchange process, KB keeps in touch with the intervention group by phone calls, chat apps, and personal visits scheduled twice a month (which can be a place other than their workplace). KB visits them individually or in three-person groups (KB and two participants). The transfer of knowledge on empathy occurs during interactions. These visits develop a friendly relationship, provide some opportunities for clarifying the aims of intervention, highlighting the role of KB, explicating new findings and their applications, and promote nurses’ competence for using empathy skills. This stage is the core of knowledge brokering intervention which is performed in parallel with other stages.

- 5. Network development, maintenance, and facilitation

To create a network, measures such as holding group sessions, creating a group on Telegram, and visiting participants in three-person groups will be taken along with other stages of knowledge brokering (as previously mentioned). Networking provides the opportunity to become familiar with each other and share their knowledge, attitude, and experiences about empathy.

- 6. Facilitating knowledge and skill development

For this purpose, KB interacts with the intervention group both individually (email, phone call, chat and in-person appointment) and in group (participating in group training sessions and being joined in the “empathy knowledge translation” Telegram group) level.

- 7. Supporting individual changes by the managers

The managers have the key role in supporting changes in nurses’ practice and knowledge exchange. The following two important interventions are performed for managers;

- 7.1 Holding three meetings with nurse managers

First meeting: It is held to sensitize managers to the necessity of developing empathic behaviors in nurses and motivate them to take part in the study. This meeting is held after posttests in the comparison group and the pretests in the intervention group. Furthermore, in this meeting, the aims of the study and the importance and positive consequences of empathy are discussed.

Second meeting: During performing the knowledge exchange, this meeting is held to present study progress, nurses’ empathy score before the intervention, results of educational needs assessment, and some explanations about the measures the managers can adopt to support nurses’ empathic behaviors in cardiac wards. In this regard, managers can take the following actions: suggesting nurses to pay attention to meta-competencies such as empathy besides clinical duties, holding educational sessions on empathy in nurses’ training program, being a role model for nurses, cooperating with university’s deputy of research to provide nurses’ access to databases and journals about empathy.

Third meeting: This meeting is held after the posttest of the intervention group to conclude the study, share opinions and experiences, re-encourage managers to support nurses’ empathic behaviors, and discuss how nurses’ empathic behaviors can improve.

- 7.2 Implementing the process of knowledge exchange

The process of knowledge transfer in the manager group is simultaneous with the intervention in the nurses’ group.

The quantity and content of all of the meetings and phone calls to the participants will be recorded by KB.

### Data management

An electronic encrypted copy of the list of participants is kept in the memory of a safe computer and will be deleted after finishing the study. The principal investigator and project manager only access to this file. The statistical consultant access to the codified and anonymous copy of this list. The questionnaires are collected and encoded anonymously. Informed consent forms are kept confidential and participants’ identity is never revealed.

### Statistical methods

The intention-to-treat analysis is performed in this study. To describe data, descriptive statistical methods such as frequency distribution tables, mean, standard deviation, median, and interquartile range are used. To examine the homogeneity of the two groups for contextual variables, independent t-test or Mann-Whitney test and chi-square test are used. To investigate whether population distribution is normal, Shapiro-Wilk or Kolmogorov-Smirnov test is used. In each group, to compare the answers before and after the intervention, paired t-test or Wilcoxon test is used. To compare the changes of each response over time, repeated measures test or marginal models is used. To compare the changes of the two groups while adjusting the effect of confounding variables, the covariance analysis model or the relative change analysis is used. For missing data imputation, the last value carried forward (LVCF) method is used. SPSS (SPSS Inc. Chicago, Il, The USA) is used for data analysis. P value below 0.05 is considered as statistically significant.

### Dissemination plan

The final report is submitted to scientific journals and presented as a lecture or poster. Furthermore, a summary of the findings is sent to the participants.

## Discussion

The aim of this study is determining the effect of knowledge brokering on nurses’ empathy with patients receiving cardiac care. Lack of empathy in clinical practices brings many negative consequences for both patients and nurses [9, 11, 29]. Knowledge brokering aims to assist researchers and final users of knowledge to reach a common language and to remove barriers to the implementation of scientific knowledge in practice [30]. To our knowledge, no study has been undertaken on knowledge brokering of empathy in cardiac nurses’ population.

Studies conducted on empathy can be criticized for using inappropriate instruments [31]. What sets this study apart from others is that it will use the Monica’s instrument that measures both cognitive and behavioral empathy [32] and is well-suited to the interventions of knowledge translation. One of the strength of the present study is selecting an experimental design which besides matching the groups and using statistical tests, allows controlling the effects of confounding variables. Random allocation leads to the normal distribution of contextual variables in the groups under investigation, reducing bias, and increasing the generalizability of the data. Probably the results of this study are generalizable for nurses working with patients who experience suffering conditions such as cancer or renal failure.

The primary limitation of the study is that simultaneous investigation of the comparison and intervention groups is impossible, because there are not enough cardiac wards in Khorramabad with limited number of nurses leading to some bias.

Regarding the ethical consideration in this research the following procedures will be respected and ensured: obtaining informed consent from research participants, explicating the aims and the advantages of conducting this research, explaining the way of using the questionnaire, complying with the principle of data protection and privacy, considering the possibility of withdrawing from the study in case of reluctance to cooperate, getting an ethics code, having a dissemination plan, publishing a training file on empathy, and reporting the study results to all participants including the comparison group, and other nurses in the study setting.

## Study status

This study is currently before data analysis stage.

## Supplementary information

**Additional file 1: Fig. 1.** Study Design.

**Additional file 2: Table 1.** Enrollment, Interventions and Assessments.

## Data Availability

The datasets used and/or analyzed during the current study are available from the corresponding author on reasonable request.

## Abbreviations

SPIRIT: Standard protocol items: recommendations for interventional trials
KB: Knowledge Broker
