# Supplementary material for: The effect of knowledge brokering on nurses’ empathy with patients receiving cardiac care: a study protocol
Source: BMC Health Serv Res. 2020 Jul 23;20:680. doi: 10.1186/s12913-020-05377-1 (PMC7376315; doi:10.1186/s12913-020-05377-1)
Supplement: Supplementary file 1 — Additional file 1: Fig. 1. Study Design. [file 12913_2020_5377_MOESM1_ESM.docx]

Fig. 1

**Analysis**

**Posttest2**

**Posttest 1**

**Pretest 1**

**Pretest**

**Intervention Group (n=50)**

**Comparison Group (n=50)**

**Enrollment, Assess for eligibility, and sampling**

**Random allocation to (n=100)**

**Posttest**

**Intervention**

**Pretest2**
