# Supplementary material for: The effect of knowledge brokering on nurses’ empathy with patients receiving cardiac care: a study protocol
Source: BMC Health Serv Res. 2020 Jul 23;20:680. doi: 10.1186/s12913-020-05377-1 (PMC7376315; doi:10.1186/s12913-020-05377-1)
Supplement: Supplementary file 2 — Additional file 2: Table 1. Enrollment, Interventions and Assessments. [file 12913_2020_5377_MOESM2_ESM.docx]

Table 1

|  | | **STUDY PERIOD** | | | | | |
| --- | --- | --- | --- | --- | --- | --- | --- |
|  | | **Enrolment**  **Allocation**  **Baseline Assessment** | **Post Allocation** | | | | **End of**  **study** |
| **TIMEPOINT** | | **T1:**  **Pretest**  **(Comparison &Intervention Groups)** | **T2:**  **Posttest 1**  **(Comparison Group)**  **at week 7** | **T3:**  **Posttest 2**  **(Comparison Group)**  **at week 14** | **T4:**  **Pretest 2 (Intervention Group)**  **at week 15** | **INTERVENTION**  **(For 6 to 12 weeks)** | **T5:**  **Posttest (Intervention Group)**  **At week 28** |
| **Enrollment** | **Eligibility Screen** | **X** |  |  |  |  |  |
|  | **Informed Consent** | **X** |  |  |  |  |  |
|  | **Random Allocation** | **X** |  |  |  |  |  |
| **Interventions** | **[Comparison Group]** (**No intervention)** | **X** | **X** | **X** |  |  |  |
|  | **[Knowledge Brokering Group]** | **X** |  |  | **X** | **X** | **X** |
| **Assessments** | **[Demographic variables] (Sex, Age,…)** | **X** |  |  |  |  |  |
|  | **[Out com Variables]**  **Empathy** | **X** | **X** | **X** | **X** |  | **X** |
|  | **[Other Variables]**  **(Years of service,…)** | **X** |  |  |  |  |  |
